# Removal of Hazardous Oxyanions from the Environment Using Metal-Oxide-Based Materials

**DOI:** 10.3390/ma12060927

**Published:** 2019-03-20

**Authors:** Ewelina Weidner, Filip Ciesielczyk

**Affiliations:** Institute of Chemical Technology and Engineering, Faculty of Chemical Technology, Poznan University of Technology, Berdychowo 4, PL-60965 Poznan, Poland; ewelina.a.weidner@doctorate.put.poznan.pl

**Keywords:** oxyanions, sorption, metal oxides, environment pollution, water purification, adsorbents, hazardous metals

## Abstract

Scientific development has increased the awareness of water pollutant forms and has reawakened the need for its effective purification. Oxyanions are created by a variety of redox-sensitive metals and metalloids. These species are harmful to living matter due to their toxicity, nondegradibility, and mobility in aquatic environments. Among a variety of water treatment techniques, adsorption is one of the simplest, cheapest, and most effective. Since metal-oxide-based adsorbents poses a variety of functional groups onto their surface, they were widely applied in ions sorption. In this paper adsorption of harmful oxyanions by metal oxide-based materials according to literature survey was studied. Characteristic of oxyanions originating from As, V, B, W and Mo, their probable adsorption mechanisms and comparison of their sorption affinity for metal-oxide-based materials such as iron oxides, aluminum oxides, titanium dioxide, manganium dioxide, and various oxide minerals and their combinations are presented in this paper.

## 1. Introduction

**Figure 1:**
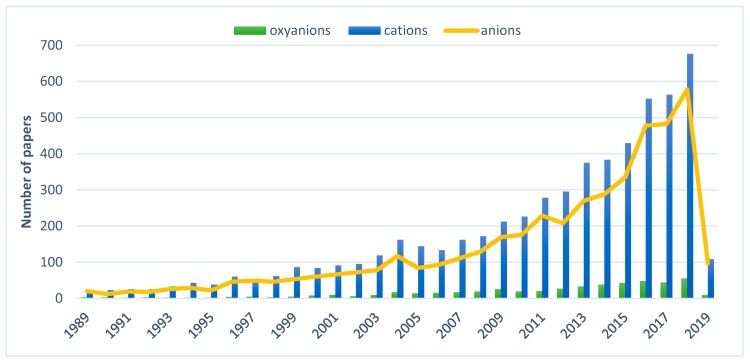
Bar chart of the number of articles per year concerning cations, anions and oxyanions adsorption for the 1989–28 January 2019. The statistical data were obtained by searching “adsorption metal oxide cations/anions/oxyanions” phrases in the Scopus data base as title and keywords.

Intensive industrial development has contributed to the increased pollution of the environment with hazardous metals and metalloids. The majority of these elements are redox sensitive and some of their oxidation states can form oxyanions in solution. Oxyanions (or oxoanions) are polyatomic negatively charged ions containing oxygen with the generic formula A_x_O_y_^z−^ (where A represents a chemical element and O represents an oxygen atom) [1]. Those compounds represent a range of different species depending both on pH and redox potential [2]. Oxyanions of As, V, B, W, and Mo are commonly found trace pollutants in various waste streams [1,3]. Metal(loid) oxyanions are characterized by toxicity [4,5,6], nonbiodegradability [5,6], and high solubility in water [7], which makes them extremely mobile harmful species which easily bioaccumulate in the environment and in the food chain [8]. Thus these species are very dangerous even at low concentrations. They can be transferred into living organisms via inhalation, ingestion, and skin adsorption, causing irreversible effects [5]. The main sources of hazardous oxyanions are alkaline wastes originating from high temperature processes with the thermal treatment of waste, fossil fuel combustion, and ferrous or non-ferrous metal smelting [2], nonetheless they are also produced in microelectronics, electroplating, metal finishing, battery manufacturing, tannery, metallurgy, and fertilizer industries [5]. However industrial activities are significantly increasing the concentration of oxyanions, and there are environments where geological formations promote dissolution of weak-acid oxyanion species, like arsenic, vanadium, or antimony, which pollute ground water sources used by local communities [9]. Considering the harmful properties of metal(loid) oxyanions, their effective elimination from water and wastewater is becoming a key issue for environment and public health protection. Nonetheless in comparison with research concerning cationic pollutants, number of works pertained to oxyanions removal is still dramatically low, what can be seen on the chart presented in Figure 1. 

**Figure 2:**
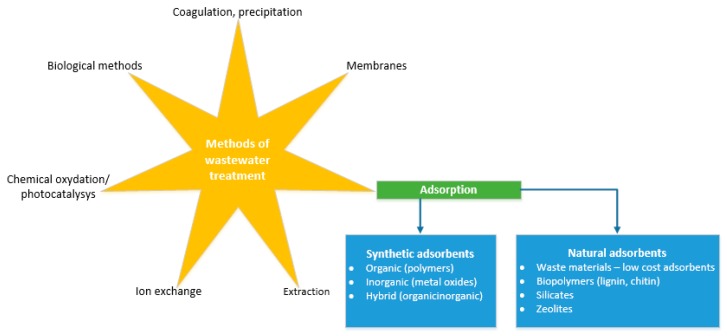
Methods of wastewater treatment.

The removal of those hazardous species from wastewater and water sources have been subjected to a variety of techniques e.g., ion exchange, filtration, adsorption, reverse osmosis, solvent extraction, chemical precipitation, evaporation and concentration, electrodialysis, and biomethods [10,11,12,13]. The main methods used for wastewater treatment with particular regard to adsorption has been shown in Figure 2.

The main problem with the majority of those methods is their high cost and need for advanced equipment. Adsorption is in the advantage over other techniques in water treatment due to its low cost, simple design, easy operation, insensitivity to toxic species, no secondary pollutants production and high efficiency [14]. Many adsorbents have been proposed in literature for water purification from harmful oxyanion species, including activated carbons [15,16], lignocellulosic materials [8], gold [17,18], silver [19], zero valent iron [20], natural minerals like zeolites [21,22,23,24], calcite [25], bentonite [14], goethite [5,26,27,28,29,30], kaolinite [31], graphene oxide [32], metal hydroxides, or metal oxides. Metal oxides are characterized by the presence of different types of surface groups [33]. Thus metal-oxide-based materials reveal a capability to adsorb ions [34] and because of that one of their major uses is for adsorbing metal or nonmetal ions from aqueous wastewater streams [35]. In this work adsorption of harmful oxyanions by metal oxide-based materials according to a literature survey have been studied.

## 2. Arsenic Oxyanions

### 2.1. Arsenic Pollution

Arsenic is a metalloid that occurs in in the +III and +V oxidation states. It is a building element of the Earth’s crust and is naturally occurring in the environment in the air, soils and rocks, natural water, and organisms [36,37]. In its inorganic form it is strongly carcinogenic and highly toxic. Nowadays arsenic pollution has been recognized to be one of the world’s greatest environmental hazards. The World Health Organization consider it one of the top ten major public health concern chemicals. The United States Environmental Protection Agency have located inorganic compounds of arsenic like arsenic acid, arsenic(III) oxide, and arsenic(V) oxide on the hazardous waste list [38]. The International Agency for Research on Cancer has classified arsenic as a human carcinogenic substance, group one [39]. Besides cancer, other negative health effects that may be associated with long-term ingestion of arsenic include developmental effects, diabetes, pulmonary disease, skin lesions, and cardiovascular disease can occur in living organisms. The current WHO recommendation of arsenic concentration in drinking water is 10 μg/L, remarking that this is only a provisional guideline arising from practical difficulties in removing arsenic from drinking water and those concentration should be as low as possible to eliminate all of its negative results. Inorganic arsenic is the most significant chemical contaminant in drinking water all over the globe. In natural waters, concentration of arsenic range from less than 0.5 μg/L to more than 5000 μg/L [37]. It is naturally present at high levels in the groundwater of a number of countries, including Argentina, Bangladesh, Chile, China, India, Mexico, and the United States of America [40]. Such high arsenic concentrations were connected to geothermal influence, mineral dissolution (e.g., pyrite oxidation), desorption in the oxidizing environment, and reductive desorption and dissolution [37]. Moreover arsenic is widely used industrially in the smelters, coal-burning, electric plants, processing of glass, pigments, textiles, paper, metal adhesives, wood preservatives, ammunition pesticides, natural weathering processes, runoff from mining operations, feed additives, and pharmaceuticals [41]. The outflow of arsenic-contaminated industrial wastewater to an aquatic system can cause deleterious effects on human health, animals, and plants. Thus elimination of arsenic from the environment is a task of high interest in research communities all over the world. Currently available techniques for arsenic removal include coagulation/precipitation, ion exchange, lime softening, reverse osmosis, electrodialysis, and adsorption. Conventionally, coagulation/precipitation with ferric and aluminum salts were used to remove arsenic from aqueous systems, but the waste sludge resulting from this process is creating problems associated with its treatment and disposal [42].

### 2.2. Characteristic of Arsenic Oxyanions in Aquatic Environment

**Figure 3:**
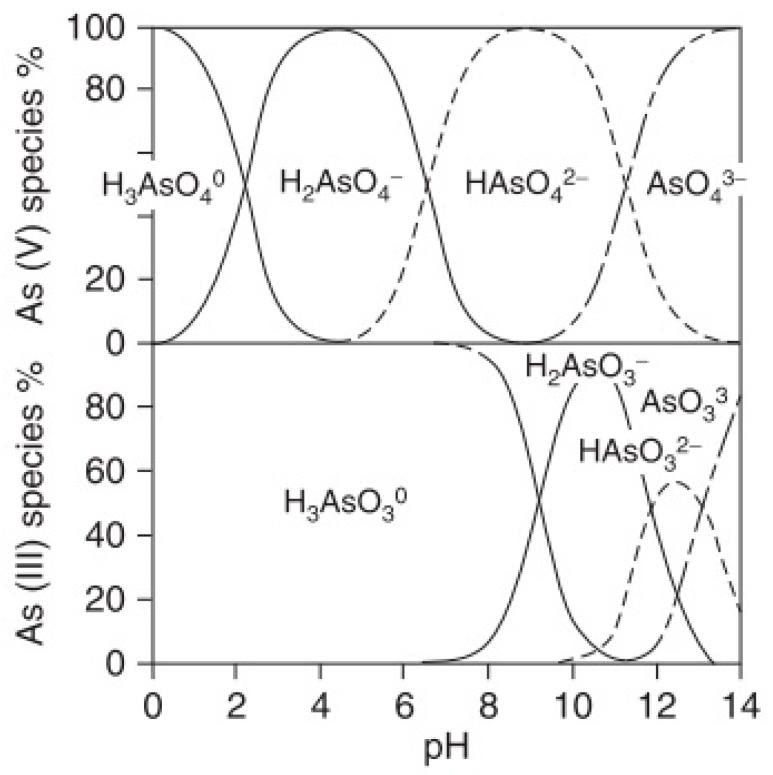
Distribution of As(V) and As(III) species as a function of pH, ionic strength = 0.04 M [43].

In the aquatic environment arsenic is able to create inorganic oxyanions—an oxidized form arsenate [As(V)] and a reduced form arsenite [As(III)] [15]. The percentage content of arsenic species in dependence with pH conditions of water is demonstrated in Figure 3. Under oxidizing conditions, arsenic usually exists in the pentavalent (arsenate) form such as H_3_AsO_4_ (dominates in the pH < 9.2), H_2_AsO_4_^−^, HAsO_4_^2^, or AsO_4_^3−^ depending on the activity of electrons (Eh) and activity of hydrogen ions (pH). Under reducing conditions arsenic mainly exist in the trivalent form (arsenite)—H_2_AsO_3_^−^ and HAsO_3_^2−^. In the typical pH for majority of natural waters (6.5–8.5) H_2_AsO_4_^–^ and HAsO_4_^2−^ are predicted to appear. The behavior of arsenic ions in the groundwater and water treatments systems is determined by the electrical charge. The strength of sorption of anions onto metal oxides is strongly dependent on the pH of the environment [36].

### 2.3. Adsorbents for Arsenic Removal from Water Sources

Adsorption technologies represent an innovative and economic approach to the arsenic removal problem. Metal oxide-based materials were successfully applied for arsenic adsorption from environment since its pollution problem was noticed. In the Table 1 sorption conditions and capacities for different arsenic species adsorbents were collected.

Aluminum oxide (Al_2_O_3_), also called activated alumina (AA), is produced by thermal dehydration of aluminum hydroxide, so that the surface can exchange contaminants for hydroxyl groups. It is characterized by a relatively high surface area (about 200 m^2^/g) and diverse pore distribution of macro and micropores. It can be regenerated with sodium hydroxide, followed by neutralization with sulphuric acid [36]. Currently it is classified by the USEPA as among the best available technology for arsenic removal in drinking water [44,45]. Activated alumina has strong selectivity to arsenate ion, is nonhazardous and can be safely disposed on landfills. Among the treatment processes for the arsenic elimination, Al_2_O_3_ adsorption is less expensive than the membrane separation, and more versatile than the ion exchange process [46]. Aluminum oxide has been widely used in West Bengal (India) [47]. Its main drawbacks are its pH sensitivity and low regeneration range of about 50–70% (must be replaced after four to five regeneration cycles) [48]. The surface of activated alumina is positively charged until the pH is lower than point of zero charge (pH_pzc_), which for different type of alumina is around 8.4–9.1 [46]. Dambies [44] in his review reported that the optimum pH value of oxyanions adsorption onto activated alumina is located in the range of 6–8, where it is predominantly positively charged, and with the increase of pH, the positive charge of Al_2_O_3_ increases, decreasing the sorption of arsenic oxyanions.

Iron oxide materials are characterized by their low cost and environmental friendliness [49]. They reveal a high affinity towards arsenic oxyanions, which makes it possible to apply them as adsorbents in water purification. An important mechanism for As(V) and As(III) removal by iron oxides is surface complexation [41]. However the most popular iron adsorbent used for arsenic removal is granular ferric hydroxide, and several iron(III) oxide materials (i.e., amorphous hydrous ferric oxide, goethite, and poorly crystalline hydrous ferric oxide) proved to be promising adsorptive materials for arsenic removal as well. Oscarson et al. [50] in 1982 investigated amounts of As(III) and As(V) adsorbed by pure Fe oxide and Al oxide. They used 0.1 g of each oxide in 70 mL of arsenic solution (adsorbent concentration 7 g/L) per 0.5–12 h. The adsorption capacities of Al_2_O_3_ were confirmed as to 16 mg/L for arsenite and 24.5 mg/L for arsenate, while for Fe_2_O_3_ 60.9 mg/g and 21.3 mg/g respectively. In their previous work they revealed that iron and aluminum oxides do not oxidize arsenite to arsenate [50]. In 2007 Jeong et al. [45] studied the adsorption of arsenate [As(V)] onto Fe_2_O_3_ and Al_2_O_3_ and they obtained significantly lower adsorption capacities using similar adsorbates concentrations—0.05–1 g/L of Fe_2_O_3_ and 0.5–6 g/L of Al_2_O_3_. The maximum adsorption capacities of Fe_2_O_3_ and Al_2_O_3_ at pH 6 were estimated from the Langmuir isotherm, and found to be 0.66 mg/g and 0.17 mg/g, respectively. However, though adsorption capacities for Al_2_O_3_ and Fe_2_O_3_ are significantly low, they are still one of the most popular adsorbents used for arsenic removal from water environment.

Lin et al. [46] investigated commercially available amorphous granular activated alumina (Macherey-Nagel, Düren, Germany) as arsenic oxyanions sorbents. Before sorption, study samples were dried in the oven at 105 ± 5 °C for 24 h and stored in a desiccator for further analysis and experiments. For better results narrow size ranges of samples were analyzed. Surface area of granular activated alumina varied from 115 to 118 m^2^/g. Arsenic sorption studies were conducted from model concentrations obtained from Na_2_HAsO_4_·7H_2_O (KR Grade, Sigma-Aldrich, St. Louis, MO, USA) and NaAsO_2_ (GR Grade, Sigma-Aldrich). Adsorption equilibrium was established within 40 h for arsenite and 170 h for arsenate and were studied for different pH and concentration of arsenic species. Obtained data fitted with both Freundlich and Langmuir isotherm equations and all the nonlinear regression coefficients were larger than 0.93 which indicated that both models successfully describe the partition behavior between water and the granular activated alumina surface for arsenite and arsenate. Davis and Misra [35] investigated the influence lanthanum oxide presence on Al_2_O_3_ adsorption properties regarding to As(V) oxyanions. An obtained hybrid oxide system containing of 10% lanthanum oxide and 90% of aluminum oxide (activated γ-alumina) revealed adsorption capacities of 0.050 mg/g for H_2_AsO_4_^−^ and 0.029 mg/g for HAsO_4_^2−^, which is a noticeable decrease in comparison with results of pure aluminum oxide. Researchers recognized optimal pH of the process close to and above the pH of the point zero charge of activated alumina, where its surface is neutral or negatively charged. Repulsion of negative ions from negative surface translates to a very low adsorption capacity obtained for their material in comparison with pure Al_2_O_3_ in slightly acidic pH. Perhaps authors could obtain better results if they had obeyed the basic laws of electrochemistry and set beneficial sorption conditions.

To take heed of cautionary notice on the use of aluminum-based compounds for water treatment published by World Health Organization in 1997 and problems with granular ferric hydroxide Manna et al. [47] synthesized crystalline hydrous ferric oxide (CHFO) and investigated its sorption properties for arsenic removal. Tests were run onto model solutions prepared from sodium metaarsenite and disodium hydrogen arsenate of A.R. grade (British Drug Houses). CHFO were prepared by hydrolysis of 0.1 M FeCl_3_ in 0.01 M HCl with 0.1 M NH_3_ solution to obtain a pH in the range of 4–5. The precipitate was aged with the mother liquor for five days, then the acid was removed and material was dried in 40 °C in an air oven. Experiments revealed that adsorption follows a first-order Lagergren kinetic model and the data fit the Langmuir isotherm. For maximum As(III) and As(V) adsorption CHO needed 3 and 5 h respectively. The increase of adsorbent drying temperature onto As(V) sorption from 25 to 300 °C resulted in an increase in the number of active sites and porosity due to removal of physically adsorbed water molecules. The optimum drying temperature for adsorption of inorganic arsenic species from natural water samples is 200 to 300 °C. The surface area of CHO had not been investigated. The regeneration of arsenic(III)-rich CHFO (As content: 66.6 mg/g) conducted by the authors revealed that a 1.0 M solution of NaOH or KOH is able to desorb ~60 ± 1% of initial arsenic content. About 15–20% of adsorbed arsenic does not desorb even in these harsh conditions, which may be the result of chemisorption or fouling of the adsorbent. Thus after regeneration CHFO will be 15–20% less effective in arsenic adsorption. A total of 99 ± 0.5% of the arsenic content was recovered from arsenic-rich regenerate, thus the solution obtained after its recovery can be discharged safely onto surface soil, which prevents its further disposal in the environment.

Arsenate and arsenite can be successfully removed by zerovalent iron (ZVI), which corrodes in water environment creating magnetite, a permeable reactive barrier of ZVI in the subsurface. This fact pushed Su et al. [51] to investigate magnetite as a sorbent for inorganic arsenic. In their research eight different magnetite types with different surface area and purity of the material were used. Reagent grade Na_2_HAsO_4_ (Aldrich) and NaAsO_2_ (Baker) were used as the inorganic arsenic source. Below pH 5.6–6.8 As(V) sorption were favored, while in a pH value above 7, As(III) was strongly attracted to the minerals. Magnetites revealed an ability to oxidize As(III) to As(V) and the oxidation range increased with increase of the pH from 2 to 12. The authors suggested the preparation of an engineered system where magnetite will be a favorable corrosion product of ZVI, which would be effective for arsenic removal.

However, though adsorption of hazardous metals is an effective removal technique, it does not cause their annihilation. Some researchers have worked on combining adsorption with other techniques to completely destroy or remold heavy metals into harmless compounds. One of the most popular transformation processes is oxidation. Photocatalytic activity of titanium dioxide was previously used in the oxidation of arsenite to arsenate. Bissen et al. [52] effectively photooxidized As(III) to As(V) using suspension of TiO_2_ in water with simulated or natural sunlight as irradiation sources. However, TiO_2_ has a low surface area and low adsorption capability, batch experiments proved that in the natural sunlight part of the arsenic was adsorbed onto it. Moreover it is very hard to remove the arsenate from contaminated water at the same time as oxidation occurs. Those facts pushed Zhang and Itoh [53] to combine titanium dioxide with iron oxide and slag (SIOT) to obtain device for arsenic removal from high-concentration arsenic contaminated wastewaters (100–20,000 mg/L). They used slag obtained from a municipal solid waste incinerator (Resource Recovery Center of Toyohashi, Aichi, Japan), TiO_2_ in an anatase form with purity 99.9% (High Purity Chemicals) and analytical grade FeCl_3_ (Wako or Aldrich). Slag (50 g) was aged for 2 days in a NaOH solution to obtain nearly neutral pH, then FeCl_3_ solution was added and aged for 12 h on magnetic stirrer and finally 5 g TiO_2_ was added. After 2 h the slurry was filtrated and dried at 105 °C for 2 h and then at 550 °C for another 1 h. Finally, the composite material has been grinded into separate grains and dried at 105 °C for 24 h under the vacuum. The obtained material’s surface area was investigated by the BET method (Quantachrome Monosorb MS-21, Boynton Beach, FL, USA) and was equal to 163 m^2^/g which was lower than material without TiO_2_ (196 m^2^/g) which was previously synthesized by the research group. The addition of 10% of TiO_2_ reduced surface area by about 20%. However, though the oxidation of As(III) to As(V) was rapid and effective, the adsorption of produced As^5+^ ions was slow. Adsorption capacity for pure TiO_2_ was 0.0001 mg/g, while for SIOT increased to 0.0047 mg/g and remained still very low. Analogical system without the slag (NHITO) was investigated by Gupta et al. [54] and they obtained slightly different results. Material was prepared in a low temperature process of slow injection of 10 g TiCl_4_ into a 0.5 M FeCl_3_ in hot 0.1 M HCl solution (~60 °C) with mechanical stirring. The pH of the mixture was regulated to 5.0–6.0 with 1 M NaOH. The formed precipitate was aged for 6 days in mother liquor, washed with deionized water till the alkali were free, dried in air oven at 60–70 °C, cooled with ice cold water, broken into agglomerated particles, and sieved for use. However, though the BET surface area of the obtained bimetal oxide was equal to 77.8 (±0.2) m^2^/g, which is much lower than for material obtained by Zhang and Itoh [53], it was characterized with a much higher adsorption capacity equal to 85.0 mg of As(III) per g of adsorbent and 14.3 mg of As(V) per g. Adsorption of arsenic species followed the Langmuir model and was favorable in pH = 7 at a temperature of around 30 °C. Adsorption of the As(III) species is not one of the key factors in arsenic removal, because those species can be easily oxidized to As(V) ones. The main problem is arsenate elimination, and for As(V) removal, NHITO is no competition for other adsorbents which are cheap and available, like crystalline hydrous ferric oxide.

Another metal oxide with the ability to convert arsenite to arsenate is manganese dioxide (MnO_2_). Manganese dioxide in its mineral form has been used in water treatment for more than 75 years, efficiently removing iron, manganese, and arsenic at pH between 5–9 [55]. Manganese oxides are very active components of natural environments, able to strongly sorb ions and participate in redox reactions. They have been identified as the primary electron acceptor in the oxidation of As(III) to As(V) by freshwater lake sediments [56]. Oscarson et al. [56] investigated arsenic oxidation by manganese dioxide and proposed mechanism of it, which can be described by five equations:

(1) $${\mathrm{HAsO}}_{2}+{\mathrm{MnO}}_{2}=\left({\mathrm{MnO}}_{2}\right)\cdot {\mathrm{HAsO}}_{2}$$

(2) $$\left({\mathrm{MnO}}_{2}\right)\cdot {\mathrm{HAsO}}_{2}+{\mathrm{HAsO}}_{2}+H_{2}O=H_{3}{\mathrm{AsO}}_{4}+\mathrm{MnO}$$

(3) $$H_{3}{\mathrm{AsO}}_{4}=H_{2}{\mathrm{AsO}}_{4}^{-}+H^{+}$$

(4) $$H_{2}{\mathrm{AsO}}_{4}^{-}={\mathrm{HAsO}}_{4}^{2-}+H^{+}$$

(5) $$\left({\mathrm{MnO}}_{2}\right)\cdot {\mathrm{HAsO}}_{2}+2H^{+}=H_{3}{\mathrm{AsO}}_{4}+{\mathrm{Mn}}^{2+}$$

The first step of the oxidation process is adsorption of arsenic species creating a layer onto the MnO_2_ surface (Equation (1)). Next HAsO_2_ is oxidized to H_3_AsO_4_ with subsequent oxygen transfer. However when the pH is equal to 7 or less, the predominant trivalent arsenic form is arsenious acid (HAsO_2_), the oxidation products dissociate forming almost equal amounts of H_2_AsO_4_^−^ and HAsO_4_^2−^ with little H_3_AsO_4_ presence at equilibrium (Equations (3) and (4)). During dissociation each mole of As(III) release 1.5 moles of hydrogen ions, which should significantly lower the pH of the system when no other reactions occur. However, after the reaction the solution pH stays close to neutral, which indicates a reaction of hydrogen ions with adsorbed HAsO_2_ on MnO_2_ surface. In such a reaction the H_3_AsO_4_ is formed and manganese is reduced and dissoluted (Equation (5)).

Despite MnO_2_ being a common and effective oxidizing agent for As(III), it is characterized by a significantly low surface area, which limits the arsenic sorption capacity of this adsorbent. To overcome this disadvantage Lei et al. [57] combined MnO_2_ with iron oxide, which is known as efficient arsenic adsorbent. Using a hydrothermal method researchers prepared a flower-like three-dimensional nanostructure Fe–Mn binary oxide and compared its arsenic adsorption properties with pure manganium oxide and iron oxides. The preparation procedure of Fe–Mn binary oxide was as follows: In 76 mL of deionized water MnSO_4_·H_2_O (0.6830 g), Fe(NO_3_)_3_·9H_2_O (1.6406 g), K_2_S_2_O_8_ (1.0868 g), and 4 mL of concentrated sulfuric acid were mixed and stirred at room temperature. The homogeneous solution was autoclaved and preheated to 110 °C for 6 h. Impurities were removed using deionized water and ethanol and then precipitates were dried at 60 °C for 8 h. Pure MnO_2_ and iron oxides were prepared in the same way except that the Fe(NO_3_)_3_·9H_2_O and MnSO_4_·H_2_O, respectively, were absent. The adsorption onto prepared materials had fitted well to the Freundlich isotherm, which suggested that the adsorption mechanism is a multilayer physisorption. The surface area was 123 m^2^/g for Fe–Mn binary oxide, 77 m^2^/g for MnO_2_, and 43 m^2^/g for iron oxides measured by an unspecified technique. The highest sorption capacities occurred to be 26.50, 23.40, and 11.22 mg/g respectively. Despite quite a high increase of Fe-Mn binary oxide’s surface area in comparison with iron or manganium oxide, arsenic sorption capacity increased insignificantly compared to iron oxides. This fact throws into question the sense of using such hybrid systems in commercial arsenic removal.

Considering the harsh conditions of waste streams containing arsenic pollution, Ren et al. [49] combined iron oxide with hydrous zirconium oxide, which is characterized by high resistance to acids, alkalis, oxidants, and reductants. The Fe–Zr binary oxide was prepared by a simple coprecipitation method at ambient temperature. Ferric chloride hexahydrate (0.05 mol) and zirconyl chloride octahydrate (0.0125 mol) were dissolved in deionized water (400 mL). During stirring pH was established to the level of 7.5 by adding sodium hydroxide (2 mol/L). The formed suspension was stirred for 1 h, aged at room temperature for 4 h, washed with deionized water, filtered, dried at 65 °C for 4 h, and crushed. The surface area of obtained material examined via BET analysis was equal to 339 m^2^/g with pore volume of 0.21 cm^3^/g. The SEM images revealed amorphous structure of obtained binary oxide. The adsorption of arsenic onto Fe-Zr binary oxide was described well by the Freundlich model. The Langmuir isotherm failed to describe the adsorption behavior, despite the adsorption capacities having been calculated from Langmuir equations and found to be equal to 46.1 mg/g for As(V) and 120.0 mg/g for As (III) at pH 7.0. Similar research was carried out by Gupta et al. [58,59]. Researchers obtained nanostructure of Fe-Zr binary oxide (NHIZO) by the hydrolysis of hot (60 °C) ferric chloride (0.18 M FeCl_3_ in 0.1 M HCl) and zirconium oxychloride (0.02 M ZrOCl_2_ in 0.1 M HCl). Then the precipitate was aged, filtered, washed with deionized water, dried at 80 °C, and treated with cold water to obtain agglomerated particles ranging in size from 140 to 290 µm. As opposed to Ren et al. [49], experimental adsorption data fit well to the Langmuir isotherm. Adsorption capacities were determined by Langmuir model and were equal 65.5 mg/g for As(II) and 9.4 mg/g for As(III). Revealed data suggests that the As(III) sorption by NHIZO is physisorption in nature, while As(V) sorption reaction with NHIZO is a chemisorption phenomenon. However the hydrolysis method is environmentally friendly compared to Fe-Zr binary oxide obtained by the coprecipitation method, and NHIZO shows much lower adsorption capacities for arsenic species. Erdoğan et al. [60] obtained a nano ZrO_2_/B_2_O_3_ oxide system which was used for arsenate ion determination in tap and underground waters. Unfortunately, the authors had not explained the advantages resulting from use of such an oxide combination. Sorption capacity was determined using the batch method (pH = 3, room temperature, Langmuir model), and it was equal to 98.04 mg/g. However, while the obtained material had revealed promising sorption properties in optimal conditions, its application in the real system had not been tested. The normal range for pH for groundwater systems is 6 to 8.5 [61], while drinking water must have a pH value of 6.5–8.5, so results obtained by the authors cannot be related to real conditions.

Kwon et al. [62] immobilized zirconium oxide on alginate beads obtaining a composite adsorbent for arsenite and arsenate removal, reaching adsorption capacity of 32.3 mg/g for arsenite and 28.5 mg/g for arsenate. They used alginate as a matrix due to its strong affinity for metal ions. Immobilization using bead encapsulation is an effective way to prevent ZrO_2_ to environment. However, though the obtained material is characterized by a satisfactory adsorption capacity for arsenic ions, the system reached an equilibrium state within 240 h and the pH_pzc_ of the obtained material was 4.3, which is much lower in comparison with conventional sorbents like activated alumina.

The adsorption of arsenic oxyanions onto metal oxide materials has been widely studied by scientists all over the world. Due to the presence of a positive surface charge onto the majority of adsorbents in low pH, arsenic sorption is favorable in acidic conditions. As can be seen from the data gathered in Table 1, a lot of experiments were performed in neutral pH, which was estimated as an optimal value, which suggest that besides electrostatic attraction, another bonding takes place in arsenic adsorption. Arsenic species are effectively adsorbed in room temperature.

## 3. Vanadium Oxyanions

### 3.1. Vanadium Pollution

Vanadium is a transition metal able to create variety of compounds on oxidation states ranging from −III to V. This element is characterized by very high solubility, which causes it to distribute widely in water, soil, crude oil and air [64]. Vanadium is a redox-sensitive element that exists mainly in oxidation states: V^5+^, V^4+^, and V^3+^ [65]. Vanadium is widely applied in industries like photography, glass, rubber, ceramic, textile, mining, metallurgy, oil refiling, automobile, and in the production of pigments and inorganic chemicals [66,67]. Such multiplicitous applications results in the production of huge amounts of vanadium polluted wastes, which are discharged into environmental waters. Vanadium has been recognized as a potentially dangerous pollutant in the same class as lead, mercury, and arsenic [68]. In cases of large accidental spills or dumping of contaminated ash, there may be major toxic effects on fauna and flora. Vanadium binds strongly to soil particles and sediments, which makes an immobile element. In European soils vanadium concentration varies between 1.28 and 537 mg/kg [69]. This element can accumulate in some plants, but not in animals. Vanadium can affect organisms via inhalation of air, ingestion of food or water, or by dermal contact. Vanadium(V) (vanadate) and vanadium(IV) (vanadyl) oxyanions can have a large effect on the function of a variety of enzymes either as an activator or inhibitor of the enzyme function [70]. Pentavalent vanadium is especially harmful to human health—it can cause damage to the respiratory, gastrointestinal, and central nervous systems and disturbs metabolism [71]. The International Agency for Research on Cancer had classified vanadium pentoxide as a possible carcinogen. Currently vanadium is on the USEPA (United States Environmental Protection Agency) Drinking Water Contaminant Candidate List (CCL3) due to its potential carcinogenic effects [68,72]. Maximum concentrations of vanadium in drinking water range from about 0.2 to 100 μg/L, with typical values ranging from 1 to 6 μg/L [73,74].

### 3.2. Characteristic of Vanadium Oxyanions in Aquatic Environment

**Figure 4:**
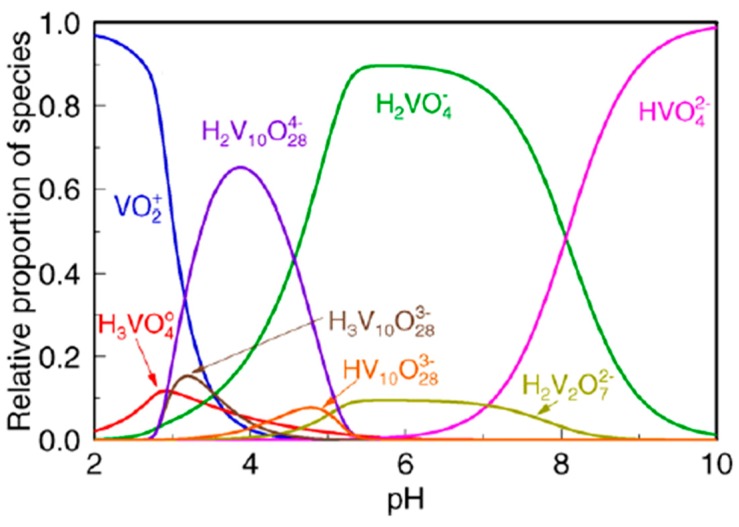
Distribution of vanadium(V) species in function of pH (initial vanadium concentration = 0.5 mM, T = 25 °C, 1 atm, ionic strength 0.15 M NaCl) taken from [74].

Baes and Mesmer [75] revealed that vanadium exists in different hydrolyzed forms depending upon its concentration and the pH of the environment. The pentavalent form is a favored state of soluble vanadium, due to V(III) and V(IV) easily undergoing rapid oxidation by a variety of oxidizing agents including air [76]. However, simple reducing agents which are frequently present in waters, i.e., oxalates, can reduce V(V) to V(IV) [71]. In an aquatic environment pentavalent vanadium occurs mostly in the presence of oxygen, creating oxyanions [72]. Twelve vanadium species can coexist in the solution [68]. Under acidic conditions (pH < 3) vanadium(V) exists in cationic form as VO_2_^+^, while in the neutral-alkaline (pH = 4–11) they occur in neutral (VO(OH)_3_) and anionic forms including decavanadate species (V_10_O_26_(OH)_2_^4−^, V_10_O_27_(OH),^5−^ V_10_O_28_^6−^) and mono- or polyvanadate species (e.g., VO_2_(OH)_2_^−^, VO_3_(OH)^2−^, VO_4_^3−^, and V_2_O_6_(OH)^3−^, V_2_O_7_^4−^, V_3_O_9_^3−^, V_4_O_12_^4−^) [72]. The specification of pentavalent vanadium forms in dependence of the environment’s pH as shown in Figure 4.

### 3.3. Oxide-Based Material for Vanadium Oxyanions Adsorption

Vanadium(IV) in contrast to vanadium(V) can be adsorbed onto various oxides and form complexes with organic matter, which makes it easily removable from the water phase into the sediment phase. Therefore, the removal of vanadium(V) from industrial wastes is of great importance for environmental protection. Nowadays vanadium is removed from water using biological, physical, and chemical techniques [76]. Adsorption, as an environmentally friendly and economic method, is one of the possible ways to do it.

Naeem et al. [68] examined vanadium adsorption onto commercially available metal oxide adsorbents currently used for arsenic removal—GTO from Dow, which is adsorbent-based on TiO_2_, E-33 from Seven Trents, and GFH (Granular Ferric Hydroxide) from US Filter, which are iron-based ones. Experimental data revealed that pH in the range of 3.0–3.5 is favorable for vanadium sorption, which is in agreement with the electrostatic attraction between protonated sites and strongly anionic metal species. Competition between hydroxide and aqueous vanadium ions for available surface sites may cause a decrease in vanadate adsorption in high pH values. At a high pH the vanadate anion can specifically adsorb in the form of HVO_4_^2−^ via a ligand exchange process, which is also characteristic for phosphate, which may suggest similar adsorption behavior of these two oxyanions.

**Figure 5:**
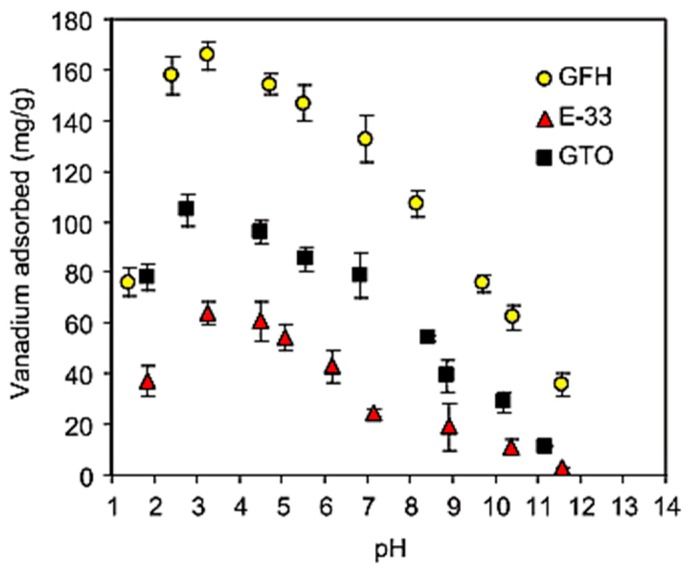
Vanadium adsorption isotherms obtained for 0.35 g/L dry mass of GFH and E-33, and 0.50 g/L of GTO (25 °C, ionic strength 0.01 M NaClO_4_, initial vanadium concentration 50 mg/L), taken from [68].

**Figure 6:**
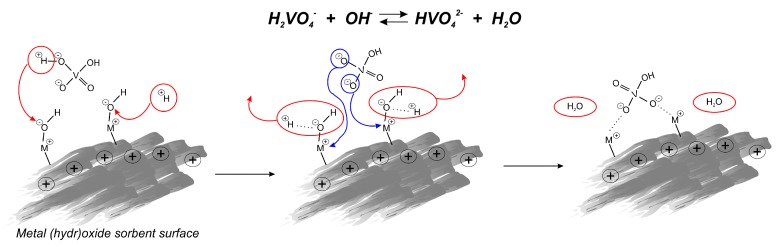
Mechanism of vanadate bonding to the surface of metal (hydr)oxide adsorbents proposed by Naeem et al. [68].

The adsorption capacities for vanadium removal increase in the following order: GTO < E-22 < GFH. GFH revealed almost four times higher effectiveness than the other iron-based adsorbent E-22, which may be a result of its higher porosity and surface area, and less crystalline, more amorphous mineralogy. However, adsorption efficiency differs significantly between tested samples, and all of them gave similar adsorption isotherm shapes (Figure 5), which indirectly confirms that the same vanadate sorption mechanism is correct for the different metal oxides/hydroxides. The uptake of vanadate on oxides/hydroxides occurs through an anion exchange mechanism including the formation of binuclear-bridged complexes. The change of initial and final pH of the system results from substituting surface OH^−^ groups with HVO_4_^2−^ ions from the solution. The hydroxide ion desorbs from the metal oxide surface being neutralized by H^+^ ions, coming from the deprotonation of the H_2_VO_4_^2−^, forming a water molecule, which is shown in Figure 6.

Leiviskä et al. [72] examined six commercial iron sorbents in vanadium removal from real industrial wastewater—commercial iron sorbent (CFH-12), commercial mineral sorbent (AQM), blast furnace sludge (BFS), steel converter sludge (SCS), ferrochrome slag (FeCr) and slag from a steel foundry (OKTO). Composition of sorbents was determined via XRF and XRD analyses and are shown in the Table 2. Experiments were carried out in batch and continuous flow pilot systems.

Firstly, all sorbents (5 g/L) were tested in the original pH (5.8) of wastewater in room temperature. Commercial iron sorbent achieved the highest vanadium reduction (73%). BFS, SCS, and AQM reached removal efficiency around 20% (27, 22, and 16%, respectively). OKTO and FeCr reveled vanadium removal efficiency below 10%. The effect of pH on vanadium sorption with sorbents CFH-12, AQM, BFS, and SCS were investigated at a fixed sorbent dosage of 10 g/L. CFH-12 was stable in a whole pH range reaching removal rates at the level of 91–94% and adsorption capacities in the range 4.7–5.1 mg/g. BFS exhibited the highest vanadium removal of 93% at low pH (4.2–5.0). For AQM and SCS sorbents the effect of pH was less pronounced, but the lowest efficiencies were observed in high pH. The CFH-12 was proven to be the most effective in vanadium removal, so the authors examined deeply only that sorbent. CFH-12 was able to reach equilibrium at the level of 10 g/L and adsorption data were fitted to the Langmuir model, which refers to monolayer sorption phenomenon [77]. The highest efficiency of that sorbent could be explained by its amorphous structure and high iron content. Amorphous iron oxides have a large surface area and hence a greater amount of sorption sites, which results in a higher adsorption capacity compared with crystalline iron materials. In comparison with other iron sorbents (Table 3) it can be seen that CFH-12 obtained a much lower sorption capacity, which might be caused by higher particle size. For iron sorbents, the pH of the environment had a significant effect on the surface hydroxyl groups’ protonation as well as for the vanadium form. In the studied pH range, vanadium exists mainly as anionic forms. The increase of efficiency in acidic pH, especially visible for the BFS sorbent, probably occurs due to the presence of higher amounts of positively charged surface groups (>Fe-OH_2_^+^) and thus electrostatic attraction between the vanadates and the surface increases. The surfaces of iron oxides generally have a net positive surface charge at acidic-neutral pH conditions (pH below the pH of point zero charge of a sorbent). Desorption of vanadium from CFH-12 was investigated to be successful—2 M sodium hydroxide was able to desorb vanadium efficiently. The recovery and renewed usage of vanadium removed from wastewaters is possible with commercial iron sorbent. The possibility to reduce its particle sizes could be tested to optimize sorption abilities.

Considering the ability of vanadium to adsorb onto other metal oxides/hydroxides and its behavior in water, Su et al. [78] established that vanadium should have similar adsorption characteristics to arsenic and selenium. As activated alumina is an inexpensive and efficient material for arsenic and selenium removal, researchers decided to test the adsorption of vanadium on its surface. Activated alumina used in this research was purchased from Tramfloc, Inc. (Tempe, AZ, USA) and was characterized with a BET surface area equal to 363 m^2^/g and a pH of point zero charge 8.8. The authors tested five different initial concentrations of vanadium to test the adsorption capacity of activated alumina. In this work authors tested also adsorption of arsenic and selenium, and they noticed that all ions act similarly, and environment pH influences maximum adsorption—it decreases in more acidic conditions and increases in more basic ones. Previous literature research indicates that arsenic adsorption is favorable in acidic pH which is in contrast to results presented in this work. Experimental data is presented only vaguely in the form of graphs, on which it can be seen that adsorbed amount varies from about 1 to about 45 mg/g in the initial vanadium concentration range of 10–493 mg/L, which is not an impressive result. Such low adsorption efficiency could be forecasted via a literature survey: in 1971 Golob et al. [79] reported that vanadium(V) can be only poorly adsorbed on activated aluminum oxide [63]. Unfortunately, data presented did not provide all derivatives to calculate adsorption capacity, which makes this research hard to follow and incomparable with other works.

Titanium dioxide is widely applied in water treatment, owing mainly to its photocatalytic properties. In the case of vanadium, its pentavalent form is not able to be further oxidized. Between 1990 and 2010 some researchers investigated adsorption of vanadium onto TiO_2_ surface in order to obtain vanadia-titania catalysts [80,81,82], which are still used for the catalytic reduction of nitrogen oxide [83]. Those researchers proceeded onto model solutions and adsorption did not occur as an effective method to do so. Nevertheless there is still a place for research on vanadium recovery from real wastewaters by sorbing it onto TiO_2_ in order to obtain V-Ti catalysts.

Activated carbon derived from various natural materials is one of the most widely used adsorbents i.e., for removal of organic pollutants [84]. It is known for its extended surface area, microporous structure, and great sorption abilities [85], however, it reveals a poor ion adsorption capacity. Thus, Sharififard et al. [66] decided to impregnate it with iron-oxide-hydroxide to increase its affinity to vanadium oxyanions and create new adsorbent for its removal from water. Commercially available activated carbon, manufactured by Norit (Amersfoort, the Netherlands) with the trade name Norit ROY 0.8, was modified via a permanganate/ferrous iron synthesis method. Optimum synthesis conditions were as follows: concentration of FeSO_4_ = 0.4 M, contact time = 24 h, and temperature = 55 °C. The obtained hybrid material was characterized by a lower surface area (777 m^2^/g) but higher vanadium adsorption capacity (119.01 mg/g) in comparison with pure activated carbon (surface area 1062 m^2^/g., adsorption capacity 37.87 mg/g). The adsorption equilibrium data fitted well with Freundlich isotherm, which suggested heterogeneous adsorption, what might be caused by the coexistence of different sorption sites, and/or different sorption mechanisms, or sorption of different vanadium species.

Carbon nanotubes (CNTs) doped with metal oxides are one of the most effective adsorbents used by researchers. Gupta et al. [12] prepared multi-walled carbon nanotubes doped with a palladium oxide (PdO-MWCNTs) adsorbent for vanadium removal. Multi-walled carbon nanotubes (MWCNTs) were synthesized by CVD method (purity N95%) from raw materials. Pure MWCNTs were treated with a H_2_SO_4_/HNO_3_ (3:1 v/v) mixture for 8 h at 10 °C at ultrasonic conditions. Then product was washed by deionized water and dried. The obtained acidic-MWCNTs powder was dispersed in water and combined with Pd(NO_3_)_2_·2H_2_O and NaOH to pH 10. The product was sonificated in an ultrasonic homogenizer and then stirred at 80 °C for 6 h and dried. Vanadium sorption was investigated by batch experiments. The optimal conditions for vanadium removal were as follows: pH = 3.0, initial vanadium(V) concentration = 60.0 mg/L, adsorbent dosage = 1.0 g/L, temperature 25 °C, and contact time 30 min. Adsorption isotherms and reaction kinetics imply that adsorption by PdO-MWCNTs could follow the Langmuir model and is a pseudo-second reaction. The highest vanadium removal efficiency of 93.7% was reached for initial vanadium concentration 60 mg/L and pH 3. Adsorbent recovery tests had not been proceeded. However, though the PdO-MWCNTs obtained high adsorption capacity for vanadium removal, their effectiveness had not been enormously high. Tests were carried only for model solutions. Thus complicated preparation and lack of information of adsorbent/adsorbate recovery and competitive adsorption of other ions strongly limit their usage.

Raw materials are gaining more and more attention from the research community in the field of adsorption. They are cost effective and highly available materials [86], which may be able to replace activated carbon adsorbents. Chitosan is characterized by the high capacity for the sorption of oxyanions, which are efficiently sorbed in acidic solutions by ionic interactions. This material was investigated in pentavalent vanadium sorption by many scientists [87,88,89,90,91]. Omidinasab et al. [67] decided to connect chitosan with magnetite to makes easier the separation after adsorption process. Besides its magnetic properties, Fe_3_O_4_ is characterized by chemical inertness, biocompatibility, non-toxicity, good thermal stability, and high surface area [92,93], which makes it a perfect material to create environmental friendly adsorbent. Chitosan was chemically extracted from chitin originating from shrimp shell wastes and then dissoluted in distilled water. Ferrous and ferric salts were co-precipitated by an ammonia solution at room temperature while the chitosan solution was slowly dripped into the mixture. Such prepared nanoparticles (Fe_3_O_4_-CSN) were collected using an external magnetic field and washed with distilled water. For testing the ability of vanadium removal the real wastewater samples originating from oil refinery were used. Fe_3_O_4_-CSN composite occurred to be very efficient—99.9% of vanadium was removed from the solution. The system reached equilibrium in the very short time of 10 min. Vanadium sorption is favorable in low temperatures and acidic pH. Kinetic data implies that the reaction was pseudo-second order, while equilibrium data fit better with the Freundlich isotherm model. Thus the adsorption onto chitosan-magnetite composite was a combination of physi- and chemisorption. Thermodynamic data revealed that the process was exothermic and spontaneous. However authors checked adsorbent only on two solutions in case of vanadium and palladium recovery, they claim that the Fe_3_O_4_-CSN composite can be used effectively for the removal of metal ions and for the treatment of real wastewaters without remarkable matrix effect. Nevertheless these conclusions seem to be too far-reaching and their confirmation requires further investigation.

A variety of metal oxides were used for purification of vanadium-contaminated water and wastewater. Similar to arsenic, vanadium is better adsorbed in acidic conditions, however, satisfactory results are also obtained in neutral conditions. A comparison of some adsorbents used by researchers for vanadate removal is shown in Table 3.

## 4. Boron Oxyanions

### 4.1. Boron Pollution and Its Behavior in Aquatic Media

**Figure 7:**
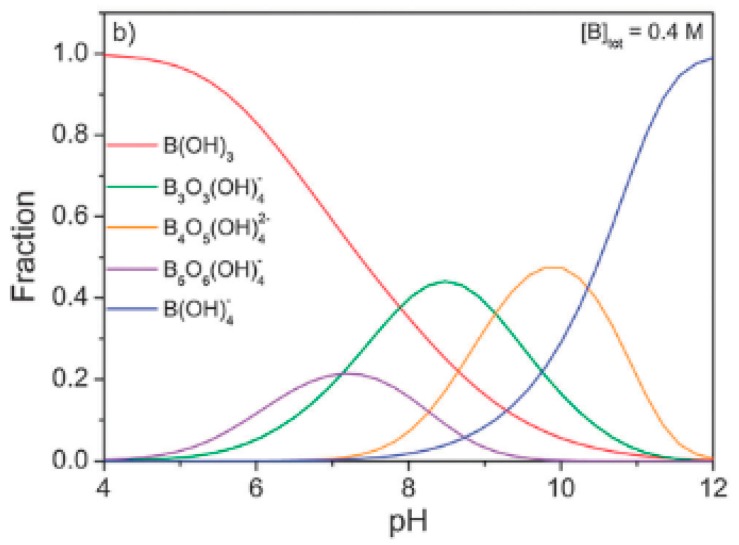
Distribution of boron species as a function of the solution pH (total boron concentration 0.4 M), taken from [101].

Boron is a metalloid that creates various compounds in five different oxidation states: −V, −I, +I, +II, and +III. Except for small amount in meteoroids, uncombined boron is never found in the elemental form in nature. Boron is a naturally found mainly as oxygen compounds (e.g., borate minerals) in oceans, sedimentary rocks, coal, shale, and some soils [95,96]. Its formation in aquatic environments is highly dependent on the hydrogen ion concentration—in pH above 8 it exists mainly as boric acid H_3_BO_3_, and below as a borate oxyanion B(OH)_4_^−^ [97]. Both of them have solution chemistries quite different from most other oxyanions. Borate is formed by the addition of a hydroxyl group to the trigonal planar boric acid molecule, creating a tetrahedral anion. In low concentrations (below 25 mmol/L) boric acid and borate exist as monomers, but with increasing concentration formation of poly-borate polymers is possible [98]. Boron species distribution is hardly dependent on the pH of the environment, as shown in Figure 7. Borates are widely used in glass production, as flame retardants, in leather production, in photographic materials, in wood preservatives and pesticides, as a high energy fuel, and in soaps and cleaners. Wastewaters polluted with boron are created mostly by glass producers and facilitate the burning of wood and coal. Boron concentration in water depends on the geochemical nature of the drainage area, proximity to marine coastal regions, and inputs from industrial and municipal effluents. In Europe, boron concentration varies from 0.001 to 2 mg/L in fresh drinking water, and similar values were reported for Russia, Turkey, Pakistan (0.01–7 mg/L), Japan (0.001 mg/L), and South Africa (0.03 mg/L). The highest concentrations were investigated in the Americas. In South America, the highest boron concentrations in boron-rich regions varied in range from 4–26 mg/L, while in other regions it was equal to 0.3 mg/L. In surface waters of North America boron concentration ranged from 0.02 mg/L to 360 mg/L in boron-rich regions, while the majority of samples were less than 0.1 mg/L [99]. The guideline value of boron concentration in drinking water was estimated as 2.4 mg/L by the World Health Organization (WHO) [100].

Waterborne boron may be adsorbed by soils and sediments and can accumulate in plants. Ingestion of high levels of boron can cause nausea, vomiting, abnormally low blood pressure, convulsions, and red lesions on the skin. Extremely low levels of boron in humans cause an increased heart rate and change of skin color to blue. High level exposure can affect the central nervous system, kidneys, and liver, and may be a leading cause of death. Borate in wastewaters is difficult to treat because it does not generate insoluble compounds with hazardous metal ions or alkaline earth metals [102]. Conventional means of water treatment (coagulation, sedimentation, filtration) are not able to remove boron completely, so more advanced and specific methods are needed to eliminate it from highly boron-polluted waters [100]. Between those methods, the most effective one is adsorption technique, due to its simplicity and the possibility to apply it in aqueous media with low concentrations of boron. Boron adsorption can be conducted on various sorbents, e.g., chelating resins, polysaccharides, synthesized clay, fly ash, and oxides [103].

### 4.2. Materials for Effective Adsorption of Boron Oxyanions

According to the literature survey made by Demetriou and Pashalidis [95], aluminum and iron oxides are the primary boron adsorption surfaces in soils, which encourage them to proceed adsorption tests using iron oxide (FeO(OH)). The adsorbent, iron-oxide (Fe(O)OH, mesh-325, Aldrich Co) was used without any further purification or other pre-treatment, while boron solutions were made from standard boron solution (99.99%, Aldrich Co) by addition of distilled water. Iron oxide’s point zero charge was reached at pH = 8. The authors investigated the optimal pH in the range of 4–12, temperature from range 20–70 °C, initial boron concentration range from 0.1–7.0 mg/L, and amount of the adsorbent range from 0.05–2.5. Optimum pH oscillates from 7 to 9 with a maximum at about 8 and it is close to the pH_pzc_ of iron oxide, and slightly lower than pKa (9.2) of the boric acid, which indicates that the best adsorption occurs when the surface has no charge and boric acid is predominant in solution. Adsorption capacity was investigated using the Langmuir model at 22 °C, and the initial boron concentration, 55 mg/L, was equal to 0.324 mg/g. The authors underline the importance of iron oxide as a boric acid adsorbent, because it affects the chemical behavior and migration of boron in the natural environment and in the purification of industrial wastewaters. It is known that iron oxide as well as activated alumina are popularly-used adsorbents for industrial wastewater treatment, not specially for boron removal. In highly boron-contaminated water, iron oxide would not be able to purify the water because of its low boron sorption capacity.

Peak et al. [104] examined hydrous ferric oxide (HFO) in boron removal obtained even worse results than Demetriou and Pashalidis [95]. The authors were not able to determine maximum adsorption capacity of HFO but the adsorbed boron amount tested for three different pHs (6.5, 9.4, and 10.4) varied from almost 0 to 160 μmol/g, which is extremely low. Moreover, adsorption isotherms did not display a particularly high affinity of boric acid for the HFO surface—to achieve a high surface loading, a high solution concentration is necessary. Except for the obtained results, the authors suggested that boric acid adsorbs via both physical adsorption (outer-sphere) and ligand exchange (inner-sphere) reactions.

Due to the need to develop alternative and cost-effective adsorbents, Irawan et al. [95] decided to test aluminum-based water treatment residuals (Al-WTRs) in boron removal. Al-WTRs consist mainly of aluminum, iron, and silica oxides with some organic compounds and they are generated from drinking water treatment facilities. Previously the Al-WTRs were investigated as anionic contaminants adsorbents i.e., fluoride (F^−^), phosphate (PO_4_^3−^), perchlorate (ClO_4_^−^), arsenate (AsO_4_^3−^), and selenium (as SeO_3_^2−^ and SeO_4_^2−^). A boron solution was prepared from analytical grade H_3_BO_3_ and adsorbents were obtained from three different water treatment plants in Taiwan. Coagulants used in water treatment plants were aluminum sulfate (sample Al-WTR1) and polyaluminum chloride (samples Al-WTR2 and Al-WTR3). Before adsorption experiments Al-WTRs were washed with deionized water to remove impurities, dried overnight in 150 °C, crushed and sieved. The chemical composition of used aluminum water treatment residuals was investigated using aqua regia-HF procedure in a Teflon closed vessel. Al-WTR1 was characterized by highest Al_2_O_3_ content (408 ± 1 mg/g), followed by Al-WTR2 (227 ± 4 mg/g) ad Al-WTR3 (150 ± 2 mg/g). Iron oxide content was similar for all samples and was equal to 195 ± 3 mg/g for Al-WTR1, 194 ± 3 mg/g for Al-WTR2 and 197 ± 2 mg/g for Al-WTR3. Silica content was highest in Al-WTR3 (528 ± 2 mg/g), followed by Al-WTR1 (376 ± 1 mg/g) and Al-WTR2 (322 ± 1 mg/g). Al-WTR1 occurred to be the best sorbent for boron probably due to its highest alumina content and highest surface area. Silica does not adsorb boron, so its role in boron removal is negligible, and surprisingly the Al-WTR3 with the highest silica content is characterized by lowest surface area. The data obtained during experiments fitted the Langmuir adsorption isotherm and the reaction rate was described as pseudo-second order model. Adsorption capacities for boron were equal to 0.98, 0.70, and 0.19 mg/g for Al-WTR1, Al-WTR2, and Al-WTR3, respectively. Authors concluded that aluminum-based water treatment residuals (Al-WTRs) can be used as alternative adsorbent for boron removal. However, it should be remembered that Al-WTRs will not be as effective as adsorbents designed specifically for boron removal because they have been designed for general water purification. Thus, Al-WTRs will not be effective adsorbents for highly boron contaminated water. To make great use from Al-WTRs in boron removal from industrial wastewaters further research to extend sorption capacity and affinity for boron compounds must be proceeded.

Adsorbents with magnetic properties are a promising technology for the future of water treatment. Such sorbents reveal the great potential of functionalization and the effective sorption of different substances [105]. Magnetic properties enable effective elimination of adsorbent from water treatment systems which simplifies its regeneration. Fe_3_O_4_-based adsorbents are characterized by large surface area and magnetic recoverability [106]. Considering the facts presented above, Liu et al. [98] tested Fe_3_O_4_ and its two composites derived from magnetite and bis(trimethoxysilylpropyl)amine (TSPA), and from magnetite and a flocculating agent 1010f (a copolymer of acrylamide, sodium acrylate, and [2-(acryloyloxy)ethyl]trimethylammonium chloride) in boron removal by means of adsorption. Pure magnetite was obtained by coprecipitation of Fe(II) and Fe(III) ions in aqueous solution with ammonia. For Fe_3_O_4_-TSPA composite, to 5.0 g of wet magnetite dispersed in water 2.5 mL of TSPA (Gelest) was added and stirred within 30 min. Composite material was removed from reaction environment using magnet and washed to inert pH. Second composite was prepared analogically, the flocculating agent was 0.5 g/L 1010f (Zibo Zhisheng Industrial Co., Ltd., PR China). For adsorption experiments 3.0 g of the wet particles were dispersed in 50 mL solution at the desired initial boron concentration, pH, and ionic strength. All of them were carried out using a SHA-C shaking water bath (Changzhou Guohua Co., Ltd., Changzhou, China) with a shaking speed of 80 rpm at 22 °C. Experimental data revealed that boron adsorption is the most favorable in pH = 6 (three initial pH were tested and adsorption decreased in order 6.0 > 2.2 > 11.7). From the boron speciation chart (Figure 6) it can be seen that researchers missed the pH range in which boron oxyanions are formed. Probably if more careful research or pH influence study had been done, the authors could have obtained much better results for their adsorbents. For all adsorbents the amount of boron adsorbed is highest in neutral solution, which may be caused by the creation of hydrogen bonding, and electrostatic and hydrophobic attractions. In alkaline conditions adsorption is the lowest, what may be the result of the electrostatic repulsion. Increasing ionic strength decreases adsorption efficiency. The highest efficiency in boron removal was observed for Fe_3_O_4_–TSPA, followed by Fe_3_O_4_–1010f, and the pure Fe_3_O_4_. The adsorption decreases with the increase in ionic strength. Adsorbents’ surfaces have the suitable functional groups and atoms for the formation of hydrogen bonds (including ionic hydrogen bonds) with boric acid and borate, which can promote the adsorption. The authors determined that three types of interactions determine boron adsorption onto Fe_3_O_4_–TSPA: (i) electrostatic interaction; (ii) hydrogen bonding; and (iii) hydrophobic interaction and proposed specific mechanism of it.

Öztürk and Kavak [107] investigated boron removal from aqueous solutions by batch adsorption onto cerium oxide. Maximum boron adsorption was obtained at original pH value of boron solution (6.18) and 40 °C by powdered cerium oxide, but the authors did not give an exact value. Experimental data were neither fitted to Langmuir nor Freundlich isotherm, which confirmed that boron adsorption onto cerium oxide is unfavorable.

In their work de la Fuente García-Soto et al. [97] investigated ability of magnesium oxide for boron removal by means of adsorption from water environment. Researchers were working on commercially available magnesium oxide produced by Panreac Chemical (Castellar del Vallès, Spain) and solutions of boric acid supplied by Merck, Darmstadt (Germany) in distilled water. Adsorption isotherms were prepared by combining growing amount of adsorbent in constant adsorbate solution in time necessary to reach equilibrium. Then the remaining boron concentration was measured. Conditions of the process were: Mg/B mol ratio, 20; stirring speed, 200 rpm; stirring time, 2 h; repose time, 48 h; room temperature; pH 9.50–10.50. Experimental data were compared using the Langmuir isotherm model. The authors studied the mechanism of boron adsorption onto magnesium oxide surface and proposed it as a three step chemisorption: (i) hydration reaction of MgO with water creating a magnesium hydroxide gel possessing active centra over the surface; (ii) alkalization due to acid–base reaction between magnesium oxide and water; and (iii) stereospecific chemical reaction between borate ions and active centers. Adsorption is an effective method of medium to high boron concentrations (50–500 mg/L) and its efficiency is set over 95% of boron removal, which is higher than natural adsorbent materials such as clays, boron containing minerals, or humic acids. This process seems to be relatively inexpensive—authors estimated the cost of highly boron-contaminated water (500 mg/L) purification at 0.96 € per m^3^. Nevertheless, this process is effective only for medium-high concentration of boron in the water and adsorbent can be used only in 3 cycles due to irreversibility of boron adsorption onto magnesium oxide.

Considering the need to design recyclable adsorbent for boron removal Kameda et al. [102] decided to investigate usage of Mg-Al oxide through the production of Mg-Al layered double hydroxides intercalated with B(OH)_4_^−^. Maximum adsorption capacity was obtained for Mg-Al oxide when the Mg/Al = 2 and was equal to 80 mg/g. Recycling of the adsorbent was proposed in two steps: (i) borate intercalated Mg-Al LDH treatment with carbonate ions in water in order to enable anionic exchange between carbonate and borate, to produce CO_3_·Mg-Al LDH, (ii) CO_3_·Mg-Al LDH calcination in 400–800 °C in order to recover Mg-Al LDH. After regeneration tests the adsorbent maintained the ability to remove boron from aqueous solution, but efficiency declined significantly—boron concentration at 480 min of adsorption for the first cycle was 32.3 mg/L, and for the regenerated adsorbent 62.1 mg/L. Such a low performance of Mg-Al oxide is connected to the decreased crystallinity and remains of adsorbed boron into the Mg-Al oxide structure. To consider the commercial use of Mg-Al oxide as boron adsorbent, the regeneration procedure needs to be refined and the contact time should be significantly reduced.

The adverse effects of boron pollution for living organisms could not be ignored. Research community developed some efficient borate sorbents based on metal oxides. Borates are preferably sorbed in alkaline pH. At pH below 8 the main boron compound is orthoboric acid and due to its lack of charge adsorbed quantity is insignificant. Thus, neutral pH could not be optimal one for boron species adsorption. The data gathered during the literature survey is shown in Table 4. However many more adsorbents were discussed in this section, only a few publications included all necessary information. 

## 5. Tungsten Oxyanions

### 5.1. Tungsten as an Environmental Threat and Its Performance in Water

Tungsten (W) is a heavy metal which creates compounds in oxidation states ranging from −4, to +6, while the most stable state is +6. In the environment, it occurs naturally in soils and sediments. In solution W is oxidized to soluble WO_4_ ions. Tungstate is able to occur in various complexes depending on the pH of the environment and total W concentration. In the range from neutral to alkaline conditions, oxyanion WO_4_^2−^ with tetrahedral coordination is the predominant form. In acidic media, tungsten aims for the creation of polymeric compound such as paratungstate (i.e., W_7_O_24_^6−^ and H_2_W_12_O_43_^10−^) with octahedral coordination [27,109,110]. Currently tungsten is classified as an “emerging contaminant” of concern by the U.S. Environmental Protection Agency (EPA) [111]. Its average concentration in the lithosphere varies in the range of 0.2–2.4 mg/kg [112]. The residence time of tungstate in the solution amounts to about 20,000 years, which is ten times longer than the time needed for ocean mixing resulting in total homogeneity of water worldwide [113]. This phenomenon causes an increase of tungsten pollution all around the world. Tungsten was believed to be inert in the environment and less-toxic than other heavy metals, so it was used in many fields of industry [27,114]. Its major uses are tungsten-cemented carbides, metal wires, turbine blades, high temperature lubricants, catalysts, incandescent lamp filaments, television sets, heat sinks, and golf clubs [110,115]. Some phosphate fertilizers may contain 100 mg of tungsten per kg. Moreover it had been treated as an alternative to lead to produce fishing weights and ammunition. After the ban on lead shot in the USA and Norway for hunting waterfowl it was used in hunting and recreational shooting. Pollution prevention program, the Green Armament Technology (GAT), developed by the US Army proposed the usage of tungsten-tin and tungsten-nylon composites as less hazardous materials for low caliber ammunition [27,114,116]. Thus in sites of firing activities, such as combat operation zones, military, commercial, and private shooting ranges, the concentration of tungsten in soil may be higher. In 2005 Strigul et al. [112] reported in probably the first paper on the treatment of tungsten as an environmental threat revealing that its levels above 1% mass basis (i.e., 10,000 mg/kg) resulted in the death of 95% of bacterial components (*Bacillus subtilis* and *Pseudomonas fluorescen*) in 3 months, and caused death of ryegrass plants and red worms. Such tungsten concentrations also inhibit the growth of bacterial colonies, what could possibly deteriorate process performance of biological wastewater treatment systems. 

### 5.2. Adsorption of Tungsten Species

Sorption processes onto the surface of minerals are crucial for regulating the distribution and mobility of trace metals in natural aquatic environments and soils, and it is hardly dependent on environmental pH. The mobility of tungstate oxyanion WO_4_ increases more in alkaline than in acidic conditions due to the occurrence of increased repulsive force between the negatively-charged mineral surface and the tungstate oxyanion [114]. Gustafsson [117] investigated tungstate and molybdate sorption onto ferrihydrite. Results indicated that both adsorptions can be described with two monodentate surface complexes in a surface complexation model, which does not exclude the existence of other surface complexes, but suggests their lower importance. Ferrihydrite exhibited a higher affinity for tungstate than molybdate and both adsorptions were strongly pH-dependent (100% efficiency of W adsorbed in pH 0–8). Iwai and Hashimoto [27] had adsorbed tungstate ions onto different clay minerals: metal oxide minerals (gibbsite, ferrihydrite, goethite, and birnessite) and montmorillonite, which is a phyllosilicate mineral. All materials were synthesized in the laboratory. To determine tungstate’s affinity for prepared adsorbents batch experiments in three different pHs—3, 6, and 9—were modeled using the Freundlich equation. Results indicate that the adsorption affinity for WO_4_ is higher for metal oxide minerals (especially for Al and Fe oxide minerals) than for montmorillonite. Generally it follows the order of Al hydroxide or Fe (oxyhydr)oxides (goethite, ferrihydrite, gibbsite) > Mn oxide (birnessite) > phyllosilicate (montmorillonite) in the whole pH range. The best adsorption capacities were obtained for acidic conditions (pH 4). Aside from adsorption capacities, researchers evaluated an influence of the presence of phosphate and molybdate oxyanions on tungstate sorption. Oxyanions of PO_4_ and MoO_4_ revealed higher affinity than tungstate in neutral-alkaline conditions. In acidic media, tungstate is more preferably adsorbed on clay minerals. Unfortunately, adsorption–desorption tests had not been conducted, so the reusability of adsorbents remains unknown. Hur and Reeder [114] investigated tungstate sorption on boehmite, which is an aluminum oxide hydroxide (γ-AlO(OH)) mineral. Boehmite occurs naturally as a common weathering product and is known as an effective sorbent for cations and anions. Adsorption was investigated during batch uptake experiments for a range of tungsten concentrations from 50 to 2000 μM, at pH 4, 6, and 8 and two different ionic strength 0.01 or 0.1 M (calibrated with NaCl). Adsorption edges exhibits the general behavior for anions, with maximum sorption in pH range 5.0–5.5. With pH increase the sorption ability decreases, with minimum values around the point of zero charge of boehmite, which is 8.6–9.1. A smaller decrease in sorption is observed at pH values below 5. Tungstate reveals a strong affinity for the boehmite surface at acidic and neutral pH. The greatest sorption of tungsten was observed at pH 4. The maximum uptake of tungstate has not been clearly determined, and adsorption capacities varied between 7.35 and 132.36 mg/g for optimal pH conditions. Lack of maxima suggest that tungstate sorption is not limited by surface site availability over the studied concentration. Desorption tests revealed that tungstate sorption is irreversible at pH 4, and slightly reversible at pH 8. However, boehmite shows good adsorption properties, but it has limited application possibilities due to its irreversible adsorption character.

Due to their large surface area and small diffusion resistance, magnetic adsorbents are of great interest. Afkhami et al. [109] investigated the effectiveness of Ni_0.5_Zn_0.5_Fe_2_O_4_ prepared according to the chemical co-precipitation method at room temperature for four different oxyanions’ removal, including W(IV). The adsorption capacity was in the order W(VI) > Cr(VI) > Mo(VI) > V(V) and for W(IV) was significantly higher than for other investigated oxyanions—72 mg/g. Desorption efficiency exceeded 98% while process was carried using 2 mol/L NaOH, as the most effective eluent and its equilibrium was reached in 15 min. Thus recovery of such adsorbents should be possible. Prepared nanomaterial can be easily dispersed in water, and due to its magnetic properties can be easily removed from the adsorption environment. The authors predict that Ni_0.5_Zn_0.5_Fe_2_O_4_ nanocomposites could be candidates for the removal of trace amounts of chromium, molybdenum, vanadium, and tungsten ions.

Due to the late discovery of tungsten’s harmful properties, only few works consider its removal via means of adsorption onto metal oxide-based materials. Researchers are at the beginning of the road to find effective sorbents for W contamination’s removal. Specific data gathered during literature studies were presented in Table 5. From the research already proceeded it is known that tungsten is favorably sorbed in acidic conditions at room temperature. In such conditions the surface of Fe, Al, and Mn oxides is positively charged, which enables tungsten oxyanions to bond via electrical attraction. However specific mechanism of tungsten adsorption still needs to be discovered and confirmed.

## 6. Molybdenum Oxyanions

### 6.1. Molybdenum Pollution and Behavior of Molybdenum Species in Water

**Figure 8:**
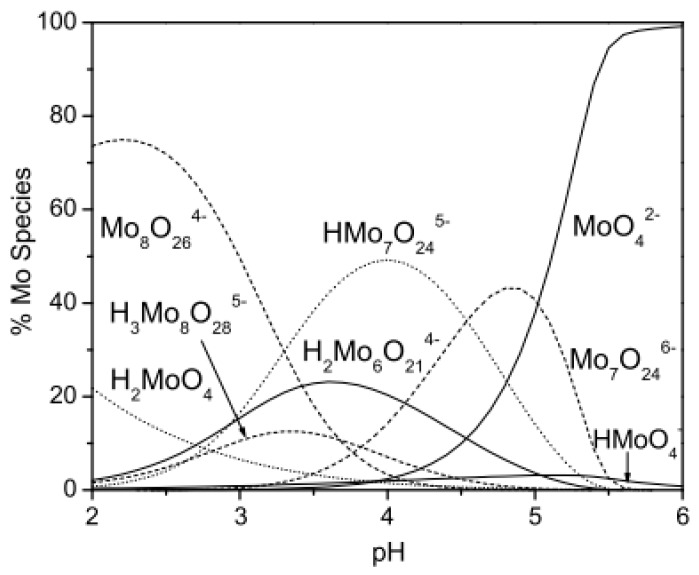
Distribution of Mo species in function of pH (initial molybdenum concentration = 10 mM), taken from [122].

Molybdenum is a transitional metal occurring on oxidation states from −II to +VI [118], which are easily convertible into each other. Naturally it is found only in minerals such as molydbenite, wulfenite, ferrimolybdite, and jordistite, which are mostly used for its commercial production [118]. Its main uses are metallurgical applications and as an alloying element in the production of stainless steel or cast-iron alloys. It is also crucial for the flame retardant, pigment, and catalyst industries [119]. The chemical properties of molybdenum exhibit similarities to tungsten and vanadium more than to chromium [118]. In an aquatic environment its main oxidation state is +VI on which it creates oxo/hydroxospecies, including polyanions. In natural waters Mo occurs mostly in inorganic forms on +V and +VI oxidation state as oxyanions such as MoO_4_^3−^, HMoO_4_^2−^, H_2_MoO_4_^−^, HMoO_4_^−^, and MoO_4_^2−^ depending on the pH of the environment [120]. In pHs on the level of 5–6 the dominant form is the molybdate anion, MoO_4_^2−^. In more acidic conditions molybdate is protonated to the less charged anionic species (HMoO_4_^−^) and in strongly acidic media the neutral molybdic acid MoO_3_(H_2_O)_3_ is created. In high molybdenum concentrations at pH below 5–6 it is able to form isopolymetalates such as Mo_7_O_24_^6−^ or Mo_8_O_26_^4−^ [121]. The distribution of molybdenum species in the function of the pH is shown in Figure 8. 

However, though its presence is essential for life on Earth, due to its ability to form active sites for many enzymes (such as xanthine oxidase, aldehyde oxidase, and hepatic sulphite oxidase [118]), that catalyze redox reactions, in high concentrations it is toxic [121,123], and may pose health problems. Subchronic and chronic oral exposure, i.e., by drinking Mo polluted water, results in gastrointestinal disturbances, growth retardation, anemia, hypothyroidism, bone and joint deformities, liver and kidney abnormalities, sterility, and also death [119]. Most molybdate compounds are harmful and toxic when injected intraperitoneally or orally in large doses (400–800 mg per kg of body weight). The WHO established 70 μg/L as a maximum molybdenium concentration in drinking water. Total molybdenum concentrations in fresh waters range from 0.03 to 475 μg/L [121]. Near the industrial sources like molybdenum mining areas its concentration in surface water can reach 200–400 μg/L, while in groundwater to even 25,000 μg/L [119]. The molybdate ion MoO_4_^2−^, as the most common oxyform of this element, occurs in many types of industrial wastewaters i.e., wastewater from a styrene monomer plant (1000 mg/L), scrubber effluent of a municipal solid waste incinerator (0.95 mg/L) and mining water (0.1–2.2 mg/L) [119]. Thus effective methods for molybdenum removal need to be discovered. 

### 6.2. Adsorbents for Molybdenum Removal from Water Environment

Adsorption as a relatively cheap and effective method for many oxyanions removal had also been considered for molybdenum oxyanions elimination. Iron, aluminum, and, to some extent, titanium oxides may be important adsorbent minerals for MoO_4_^2−^, as they may acquire positive charge at low pH [123].

Goldberg et al. [124] were interested in molybdenum adsorption onto Al and Fe oxide minerals and their adsorption mechanisms. Molybdenum adsorption on oxides exhibited maximum near pH 4 and iron oxides were more efficient adsorbents than aluminum oxides. Experimental data were fitted well to the constant capacitance model, which assumes an inner-sphere adsorption mechanism. Adsorption of Fe oxides based on a weight increased in the order: hematite < goethite < amorphous Fe oxide < poorly crystalline goethite, while for Al oxides: δ-Al_2_O_3_ < gibbsite < amorphous Al_2_O_3_. Adsorption of molydbdeum oxyanions onto aluminum oxide was a subject of research of many scientists [125,126,127,128,129] due to application of Co-MO-Al_2_O_3_ catalysts for hydrotreatment of petroleum fractions. Luthra and Cheng [126] examined molybdates (Mo_7_O_24_^6−^ and MoO_4_^2−^) onto γ-alumina via Molybdenum-95 NMR Study. Both oxyanions were attracted to the positively charged alumina surface in pH lower than the isoelectric point of the alumina (pH 8.5). NMR studies showed that Mo_7_O_24_^6−^ after the contact with alumina decomposes to MoO_4_^2−^ in minutes. The decomposition was believed to be caused by the increase in the pH of the impregnation solution inside the pores of the alumina. Wu et al. [128] investigated competition adsorption for oxyanions of similar affinity to the γ-Al_2_O_3_ surface: molybdate, selenite, selenate, chromate, and sulfate. Specific surface area of obtained γ-Al_2_O_3_ was measured by BET method and was equal to 100 m^2^/g. Molybdate and selenite are both strongly binding adsorbates—molybdate depresses selenite sorption at acidic pH and selenite suppresses molybdate sorption at alkaline conditions. The affinity for aluminum oxide surface was the highest for molybdate oxyanions, followed by selenite, selenate, sulfate, and chromate, respectively.

Xu et al. [33,123] investigated the influence of presence of phosphate, sulfate, silicate, and tungstate anions on the adsorption of molybdate onto goethite under anoxic conditions. Experiments were conducted in a glove bag (N_2_), which maintained oxygen concentration on the 1 mg/L level. Goethite slurry was synthesized by the researchers. Results indicated that MoO_4_^2−^ adsorption on goethite obeys the Langmuir model. Adsorption capacity of MoO_4_^2−^ on goethite amounted 25.9 mg/g at pH 4. Competition tests revealed that an affinity for goethite follows the order phosphate > tungstate > molybdate > silicate > sulfate. Adsorption of Mo seems to be more affected by the presence of the phosphate than the tungstate anion, which is attributable to increased repulsion between Mo and more negatively charged surface sites after phosphate adsorption onto goethite surface. The goethite surface probably contains adsorption sites common for Mo, P and W anions, as well as specific for each element. Gustafsson [117] examined molybdate and tungstate adsorption to ferrihydrite synthesized by author. However, though tungstate revealed a higher affinity for ferrihydrite, Mo oxyanions were sorbed effectively. Molybdate sorption was strongly pH dependent—the best efficiencies were obtained for acidic pH, which correlates with previous research.

Iron(II, III) oxide (Fe_3_O_4_) were used in water purification due to its magnetic properties. The main drawback of magnetite usage is its dissolution in water. High iron concentrations are toxic for humans and other living organisms. Consider that Keskin [120] decided to use an Fe_3_O_4_-embedded 1,3,5-triacryloylhexahydro-1,3,5-triazine-acrylamide hybrid polymer for molybdenum oxyanions removal. Triazine compounds are able to create complexes with iron ions which connected with polymeric network prevent iron release. The triazine molecules were used as a crosslinker and the polymeric material was synthesized by free radical polymerization. Adsorption tests were performed for pure Fe_3_O_4_, Fe_3_O_4_ embedded hydrolyzed, and nonhydrolyzed polymers. The highest removal efficiency was obtained at pH 2.5 and pure magnetite was subtly more effective than both composite materials (97% for Fe_3_O_4_ and 96.87% and 96.36% for hydrolyzed and nonhydrolyzed polymers). The iron release between those three materials varied insignificantly—the highest value was noted for magnetite—1.16 mg/L, followed by magnetite embedded by nonhydrolyzed polymer—1.02 mg/L, and the lowest value of 0.22 mg/L characterized magnetite-embedded by hydrolyzed polymer. Kinetic studies and isotherms were investigated only for an Fe_3_O_4_-embedded hydrolyzed acrylamide -1,3,5-triacryloylhexahydro-1,3,5-triazine polymer. The experimental data was fitted well to the pseudo-second-order kinetic model and Langmuir isotherm. Maximum Langmuir adsorption capacity was equal to 0.213 mg/g. Author performed reusability tests of obtained material by HNO_3_, CaCl_2_, NaOH, EDTA, HCl, and HCl/HNO_3_ solutions. The best results were obtained by CaCl_2_ treatment—sorption efficiency for molybdenum removal after regeneration increased to 97%, while its mass decreased from 0.25 g to 0.21 g, which throws into question if the adsorbent surface was chemically modified by the regenerating medium.

An innovative, attractive, and economical approach for oxyanion removal is the usage of sorbent materials consisting of a matrix and a proper adsorbent. Verbinnen et al. [119] decided to test zeolite-supported magnetite for the removal of molybdenum. The material was supposed to combine good sorption affinity for oxyanions (magnetite) and high cation affinity for cations (zeolite matrix). The composite was obtained by precipitating magnetite onto zeolite surface from Fe(II) and Fe(III) salts, and the final magnetite to zeolite ratio was equal to 1:2. A molybdenum model solution was prepared by dissolving Na_2_MoO_4_·2H_2_O in ultrapure water. Sorption was the most effective in a strongly acidic environment (pH = 3), where maximal adsorption of MoO_4_^2−^ and minimal adsorbent dissolution (Fe concentrations–0.34 mg/L, Al concentrations—0.012 mg/L). The adsorption capacity for molybdenum in optimal conditions (pH = 3 and 25 °C) is 17.9 mg per gram of adsorbent. In the absence of molybdenum the point zero charge of the obtained composite lies around the pH = 4, so below that value its surface is charged positively. In the presence of Mo the point zero charge is no longer observed and magnetite-zeolite surface is negatively charged into whole studied pH range (2–10). That facts indicated a chemical adsorption of MoO_4_^2−^, which causes the shift of pH_pzc_ to a lower value. Negatively charged Mo species (HMoO_4_^−^ and MoO_4_^2^) are pulled toward composite surface via electrostatic forces and bind chemically to the Fe(III) present on magnetite surface. Such a connection results in a negative charge of the composite, even below pH 4, and lowering of the zeta potential. In pH 4–6.5 where the composite surface is negatively charged, the attractive specific sorption forces still overcome the repulsive electrostatic forces, which indicate that molybdenum adsorption onto the magnetite-zeolite surface is a specific chemisorption process. The maximum adsorption capacity increases from 13.6 mg/g at 4 °C to 20.2 mg/g at 40 °C, which indicates the endothermic adsorption. Kinetic data fits well to pseudo second order equation. Experimental results indicate that Mo adsorption is better fitted by the Langmuir isotherm, which indicates chemical bonding between the adsorbent and adsorbate, assuming the formation of a Mo monolayer onto the magnetite-supported zeolite surface. In conclusion, the molybdenum is adsorbed onto the magnetite-supported zeolite composite via formation of an inner-sphere FeOMoO_2_(OH)·2H_2_O complex. Such a sorption mechanism was verified for different iron oxides, like goethite and feriihydrite, by Goldberg et al. [124] and Gustafsson [117]. In their later research Verbinnen et al. [130] had tested magnetite supported zeolite sorption abilities in real industrial wastewater coming from the wet treatment of flue gases from a rotary kiln for industrial waste combustion. The samples were obtained from Indaver, a waste treatment company in Belgium. However, though cations were removed by coagulation and flocculation, and mercury by precipitation with trimercaptotriazine (TMT) by Indaver, the effluent contained oxyanion-forming elements (like Mo and Sb). Anions like chloride and sulphate are hardly affected by the precipitation/flocculation treatment. Researchers increased pH from 1 to optimal sorption value—3.5. The initial concentration of Mo oxyanions was 872 μg/L and after treatment it decreased to 7 μg/L while the adsorbent concentration was 20 g/L. The adsorption order on zeolite-supported magnetite is Mo(VI) > Sb(V) > Se(VI) in both synthetic and real systems. 

Molybdenum sorption onto metal oxide-based adsorbents has not been a subject of research of many scientists. Adsorption data gained by literature survey is gathered in Table 6. Adsorption of molybdates is favored in acidic pH in room temperature. 

## 7. Conclusions

In spite of the fact that metal and metalloid oxyanion pollution is a real phenomenon and their elimination is the subject of increased interest of scientific communities, due to the lack of data in published papers the results are incomparable to each other. Authors are unwilling to share exact results of their work, which cause replication of the same studies over and over again by different research teams. In addition most of the research is carried out only on model solutions, so proposed methods might not be applicable as such for the removal of oxyanions from industrial wastewaters, because they are more complex systems containing other oxyanions and compounds, that can compete and interact with each other. Some research in the field of competitive adsorption was held, but the exact mechanisms of oxyanion behavior in solution are still unknown. Thus single sorbate and single mineral adsorption studies in the laboratory may not be directly applicable [131].

**Figure 9:**
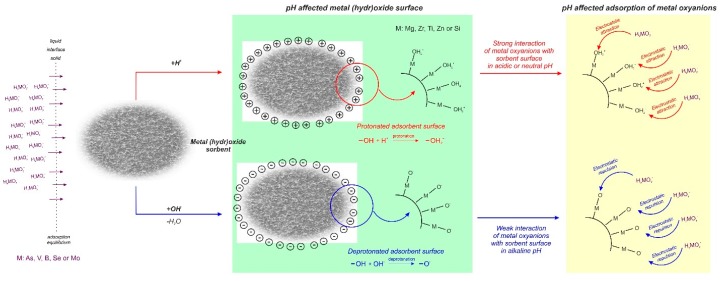
Mechanism of metal oxyanions adsorption onto metal (hydr)oxide-based sorbents.

The presented literature review unambiguously indicates the complexity of the process of removing metal oxyanions from aqueous systems via adsorption. In contrast to metal cations, which have been extensively studied and described in scientific papers, oxyanions studies are not entirely clear. Analysis of the M^n+^ ion removal process indicates the optimal conditions for its realization, which depend on many factors such as the type of cation, the type of sorption material and its physicochemical parameters, as well as the parameters of the adsorption process. The key element seems to be the pH of the adsorption environment—for metal cations, in most cases analyzed, the optimal pH at which the highest removal efficiency is observed is pH = 5–6. This is related to the Pourbaix diagrams, indicating pH, beyond which precipitation of appropriate forms of metal hydroxides will occur. In this aspect, one should also remember the influence of pH on the nature of the sorbent functional groups, which can be protonated (at low pH values) or deprotonated (at higher pH values). This is important when defining the adsorption mechanism, which in the case of M^n+^ is based on electrostatic interactions—attraction when the charge of functional groups is different from the M^n+^ charge or repulsion when these charges are the same. In this respect, the oxyanion adsorption is definitely more complex. The probable mechanism of oxyanions adsorption on the surface of (hydr)oxides has been presented in Figure 9.

**Figure 10:**
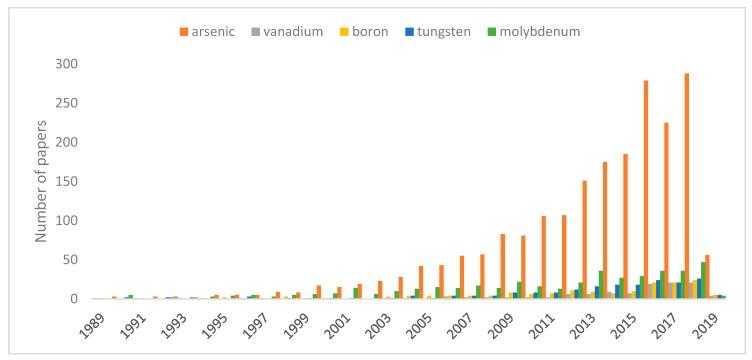
Bar chart of the number of articles per year about oxyanions adsorption for the 1989–28 January 2019. The statistical data was obtained by searching “adsorption metal oxide arsenite and arsenate/vanadate/borate/tungstate/molybdate” in the Scopus data base as title and keywords.

Moreover, a significant majority of papers pertain to arsenic removal, which is visible on the statistic presented in Figure 10. In comparison with arsenite and arsenate, research related to other oxyanions is infinitesimal and so is its significance in scientific work. 

The environment pH influences as well metal chemistry in solution (occurrence of various oxyforms) as the metal (hydr)oxide surface’s protonation/deprotonation [68]. Thus in most cases it is the determining factor of adsorption effectiveness, but unfortunately for research results it is neglected. 

The spectrum of speciation forms of oxyanions of the relevant metal is very wide, which translates into their random behavior in aqueous solutions, especially those with varying pH. Unfortunately, the aspect of the effect of pH on the efficiency of oxyanion-binding by various sorbents, e.g., those presented in the literature review, is treated fairly generally, and in some cases even overlooked. This hinders the interpretation of the dependencies obtained and introduces confusion regarding the comparison of the behavior of various sorption materials towards the removal of these types of impurities from aqueous solutions. The presented comparisons unequivocally do not indicate optimal conditions for the removal of a particular oxyanion group using the available sorption materials. It seems that the mechanism of their binding should be at least similar for different sorbents, and as a result it is completely random. Analyzing at least the effect of the pH discussed earlier, one would expect such an environment pH, which would enable a strong attraction between the positively charged surface of the sorbent and the negative form of most metal oxyanions. On the other hand, paying attention to the type of sorption material, it would be important to use one which after synthesis or preparation would exhibit a significant positive charge that could ensure strong interaction with oxyanions. These issues should not be problematic when conducting research on model solutions, which are different to real wastewaters as their composition is diverse (high concentrations of components and their diversity) and can affect the selective adsorption of individual components. All oxyanions presented in this review, excluding borates, are preferably adsorbed in acidic media, due to the positive electrical charge present on adsorbents’ surfaces. Electrostatic attraction is one that the most important mechanism of oxyanions bonding.

The following issues seem to be worth analyzing here:

- The role of sorption material, and in fact its physicochemical parameters designed at the synthesis stage—the presence of functional groups exhibiting a positive charge, facilitating the binding of negatively charged oxyanions;
- The influence of the pH of the adsorption environment on the nature of functional groups of the sorption material and the form of oxyanions in aqueous solutions, so important analyzing their potential interactions;
- Selectivity tests of sorption materials towards various metal oxyanions, which in the scientific papers published so far are effectively omitted, inversely as in case of sorption of metal cations,
- The effect of the presence of other components of wastewater on the sorption efficiency of a particular oxyanion group.

In these aspects, the presented scientific papers leave considerable dissatisfaction, and on the other hand, they open a wide range of activities to optimize the process of removal of these particularly “uncomfortable” inorganic impurities present in water systems. This all is more justified when analyzing the number of works published on this subject.

## Figures and Tables

**Table 1:** Sorption properties of metal oxide-based adsorbents for arsenic oxyanions removal.

Adsorbent	Surface Area (m^2^/g)	As Concentration (mg/L)	Adsorption Capacity (mg/g)		Temperature (°C)	Contact Time (h)	pH	Ref.
			As^3+^	As^5+^				
Al_2_O_3_	-	100	16.0 ± 0.9	24.5 ± 1.6	25	12	7.0	[56]
	0.55	0.6	-	0.14	23 ± 0.5	2	7.0 ± 0.1	[45]
	0.55	0.2	-	0.098	25 ± 0.5	1–2	6 ± 0.1	[63]
Al_2_O_3_ (granular)	115–118	0.79–4.90	1.69	-	25 ± 0.5	40	6.1 (±0.1)	[46]
	115–118	2.85–11.50	-	15.90		170	5.2 (±0.1)	[46]
Al_2_O_3_-La_2_O_3_	-	0.51	-	0.050	21	48	7.8–9.3	[35]
	-	3.62	-	0.029	21	48	7.8–9.3	[35]
Fe_2_O_3_	-	100	60.9 ± 1.1	21.3 ± 0.1	25	12	7.0	[56]
	5.05	0.6	-	0.56	23 ± 0.5	1	7 ± 0.1	[45]
	5.05	0.2	-	0.616	25 ± 0.5	1–2	6 ± 0.1	[63]
Crystalline hydrous ferric oxide	-	50	66–68	55–58	30 ± 2	4	7.0	[47]
Fe_3_O_4_ (magnetite)	2.43–16.5	2	0.65	-	-	24	7.0	[51]
	2.43–16.5	2	-	0.7	-	24	2.5–4.0	[51]
TiO_2_	-	100	0.0001	-	40	10	3.0	[53]
Slag-Fe_2_O_3_-TiO_2_	163	100	0.0047	-	40	10	3.0	[53]
Fe_2_O_3_-TiO_2_	77.8 ± 0.2	5–10	85.0	14.3	30 ± 2	3.5/6	7.0 ± 0.1	[54]
MnO_2_	77	60	2.55 (As^3+^ + As^5+^)		22	1/6	4.0	[57]
Fe_2_O_3_-MnO_2_	123	60	9.89 (As^3+^ + As^5+^)		22	1/6	4.0	[57]
Fe_2_O_3_-ZrO_2_	339	5–40	120.0	46.1	25 ± 1	36	7.0 ± 0.1	[49]
	-	10	66.5 ± 1.8	-	30 ± 1.6	2	7.0 ± 0.2	[58]
	263	10	-	9.36	30 ± 1.6	1.6	7.0 ± 0.2	[59]
Nano ZrO_2_-B_2_O_3_	-	5–300	-	98.04	room	2	3.0	[60]
ZrO_2_-alginate beads (ZOAB)	13.2	32.9	32.3	-	25	240	~5.0	[62]
	13.2	35.2	-	28.5	25	240	~5.0	[62]

**Table 2:** Iron sorbents used for vanadium removal by Leiviskä et al., reproduced from [72]. Commercial iron sorbent (CFH-12), commercial mineral sorbent (AQM), blast furnace sludge (BFS), steel converter sludge (SCS), ferrochrome slag (FeCr), and slag from a steel foundry (OKTO).

Material	XRF Results	XRD Results
**CFH-12**	83% FeO, 6.1% S, 4.2% MgO, 1.4% SiO_2_, 1.1% CaO	Gypsum (CaSO_4_·2H_2_O) mostly amorphous iron material
**AQM**	40.1% SiO_2_, 24.8% Al_2_O_3_, 18.3% FeO, 3.4% MgO, 2.9% K_2_O	Quartz (SiO_2_) Muscovite (KAl_2_(Si_3_Al)O_10_(OH, F)_2_) Kaolinite (Al_2_Si_2_O_5_(OH)_4_)
**BFS**	63.2% FeO, 12.5% CaO, 11.0% SiO_2_, 2.9% Al_2_O_3_, 2.2% MgO, 1.0% K_2_O	Hematite (Fe_2_O_3_) Calcite (CaCO_3_) Quartz (SiO_2_)
**SCS**	90.3% FeO, 5.0% CaO, 1.4% SiO_2_ 56.3%	Magnetite (Fe_3_O_4_) Hematite (Fe_2_O_3_) Cuspidine (Ca_4_(F_1.5(_OH)_0.5_)Si_2_O_7_)
**OKTO**	56.3% CaO, 26.6% SiO_2_, 6.6% MgO, 3.1% F, 2.3% Al_2_O_3_, 1.3% Cr_2_O_3_	Periclase (MgO) Calcium hydroxide (Ca(OH)_2_) Enstatite (Fe_0.3_Mg_0.7_SiO_3_) Calcium silicate (Ca_2_SiO_4_)
**FeCr**	32.5% SiO_2_, 25.8% Al_2_O_3_, 24.1% MgO, 11.2% Cr_2_O_3_, 4.3% FeO, 1.4% CaO	Spinel magnesioferrite Aluminum iron oxide (AlFe_2_O_4_) Iron silicon oxide Chromium iron (Cr_0.7_Fe_0.3_) Magnesium aluminum chromium oxide (Mg(Al_1.5_Cr_0.5_)O_4_)

**Table 3:** Sorption properties of metal oxide-based adsorbents for vanadium oxyanions removal.

Adsorbent	Surface Area (m^2^/g)	V Concentration (mg/L)	Adsorption Capacity (mg/g)	Temperature (°C)	Contact Time (h)	pH	Ref.
GFH (584 mg Fe/g GFH)	231	1–250	111.11	25	24	7.0 ± 0.1	[68]
E-33 (574 mg Fe/g E-33)	128	1–250	25.06	25	24	7.0 ± 0.1	[68]
GTO (650 mg Ti/g TiO_2_)	150	1–250	45.66	25	24	7.0 ± 0.1	[68]
CFH-12	173	58.2	5.71	room	24	5.8	[72]
AQM	-	58.2	1.72	room	24	5.8	[72]
BFS	-	58.2	1.93	room	24	5.8	[72]
SCS	-	58.2	2.62	room	24	5.8	[72]
Fe-AC	777	25–200	119.01	25	24	4.5	[66]
CeO_2_/CuFe_2_O_4_	190.2	30–250	798.6	25	3	6.0	[94]
Fe_3_O_4_-CSN	35.6	16.37	186.6	19.85	1/6	5.0	[67]
PdO-MWCNTs nanocomposites	209.59	60	245.05	25	0.5	3.0	[12]

**Table 4:** Sorption properties of metal oxide-based adsorbents for boron oxyanions removal.

Adsorbent	Surface Area (m^2^/g)	B Concentration (mg/L)	Adsorption Capacity (mg/g)	T (°C)	Contact Time (h)	pH	Ref.
MgO	-	50	303.87	room	48	9.5–10.5	[97]
	-	500	542.11	room	48	9.5–10.5	[97]
FeO(OH)	-	55	0.324	22 ± 3	-	8	[95]
Al_2_O_3_-Fe_2_O_3_-SiO_2_ (Al-WTR1)	40.5 ± 5	5–100	0.980	room	24	8.3 ± 0.2	[108]
Al_2_O_3_-Fe_2_O_3_-SiO_2_ (Al-WTR2)	34.6 ± 3	5–100	0.700	room	24	8.3 ± 0.2	[108]
Al_2_O_3_-Fe_2_O_3_-SiO_2_ (Al-WTR3)	14.5 ± 1	5–100	0.190	room	24	8.3 ± 0.2	[108]
MgO-Al_2_O_3_	-	108–648	80.00	30	168	10.5	[102]

**Table 5:** Sorption properties of metal oxide-based adsorbents for tungsten oxyanions removal.

Adsorbent	Surface Area (m^2^/g)	W Concentration (mg/L)	Adsorption Capacity (mg/g)	Temperature (°C)	Contact Time (h)	pH	Ref.
Ni_0.5_Zn_0.5_Fe_2_O_4_	-	10–250	72	25	0.5	5	[109]
Boehmite (γ-AlO(OH)	136	1000	7.35–132.36	room	24	4	[114]
Birnessite (MnO_2_)	-	18–359	6.15	25	24	4	[27]
Ferrihydrite (Fe_2_O_3_)	-	18–359	30.24	25	24	4	[27]
Gibbsite (Al(OH)_3_)	-	18–359	49.82	25	24	4	[27]
Goethite (α-FeO(OH))	-	18–359	43.12	25	24	4	[27]

**Table 6:** Sorption properties of metal oxide-based adsorbents for molybdenum oxyanions removal.

Adsorbent	Surface Area (m^2^/g)	Mo Concentration (mg/L)	Adsorption Capacity (mg/g)	Temperature (°C)	Contact Time (h)	pH	Ref.
Fe_3_O_4_ embedded hydrolyzed triazine polymer	-	2.5	0.213	25	2.5	2.5	[120]
zeolite-supported-Fe_3_O_4_	74.5	1	17.92	25	24	3	[119]
Goethite	-	1	1.76	25	24	3	[119]
	43.96	0–32	25.9	room	17	4.0 ± 0.1	[33,123]
Hematite	-	1	1.43	25	24	3	[119]
